# The hidden dangers of cerebral venous sinus thrombosis: insights into maternal morbidity and mortality during pregnancy

**DOI:** 10.3389/fmed.2026.1790485

**Published:** 2026-07-09

**Authors:** Jizi Shen, Zhongyi Gu, Li Liu, Yina Wu, Li Li, Rui Guan

**Affiliations:** 1 Department of Obstetrics and Gynecoloay, Changhai Hospital, Naval Medical University, Shanghai, China; 2 Stroke Center Neurosurgical Wards, Changhai Hospital, Naval Medical University, Shanghai, China

**Keywords:** cerebral venous sinus thrombosis, pregnancy, prognosis, recurrence, risk factor

## Abstract

**Background:**

Cerebral venous sinus thrombosis (CVST) is a rare but potentially fatal neurological emergency during pregnancy and the puerperium. Data on its prognosis in early pregnancy and the risk of recurrence in subsequent pregnancies remain limited, particularly in Chinese populations. This study aimed to identify risk factors associated with the occurrence and prognosis of pregnancy-associated CVST and to evaluate the outcomes of subsequent pregnancies in affected women.

**Methods:**

We conducted a retrospective case–control study at a tertiary referral center in China from January 2010 to June 2024. The study included 20 consecutive pregnant or postpartum women diagnosed with CVST and 198 controls without CVST. Clinical characteristics, laboratory indicators, neuroimaging findings, and treatment outcomes were compared between groups. Surviving patients were followed up for a median of 90.6 months to assess subsequent pregnancy rates and CVST recurrence.

**Results:**

Of the 20 CVST patients, 7 (35.0%) occurred during early pregnancy and 13 (65.0%) in the postpartum period. The early pregnancy group had higher rates of intracerebral hemorrhage (85.71% vs. 7.69%), cerebral infarction (57.14% vs. 30.77%), and mortality (42.86% vs. 7.69%) compared with the postpartum group. Lower apolipoprotein A1 (APO-A1) levels were associated with CVST occurrence (OR = 0.005, 95% CI: 0.000–0.158, *p* = 0.003). During follow-up, 3 of 16 surviving patients (18.75%) had subsequent pregnancies, and no CVST recurrence was observed.

**Conclusion:**

Early pregnancy CVST is associated with a more severe clinical course and higher mortality than postpartum CVST, warranting vigilant monitoring. Lower APO-A1 levels may serve as an associative marker for CVST risk. Based on our limited data, a prior history of CVST may not be an absolute contraindication to future pregnancy, though individual risk assessment and multidisciplinary management are essential.

## 1 Introduction

Cerebral venous sinus thrombosis (CVST) is a rare neurological emergency, with an estimated incidence of approximately 5 per million individuals in the general population (1). Women are disproportionately affected compared to men, largely due to gender-specific risk factors including oral contraceptive use, pregnancy, the puerperium, and hormone replacement therapy (2). Pregnancy-associated CVST is defined as CVST occurring during gestation or within the first 6 weeks postpartum. Although CVST accounts for only approximately 1.00% of all pregnancy-related strokes, it is associated with substantial maternal morbidity and mortality, posing significant threats to both maternal and fetal wellbeing (3).

The clinical presentation of CVST is highly heterogeneous, often leading to diagnostic uncertainty and delayed treatment, and reliable early prognostic markers remain lacking (3, 4). Although previous studies have described the overall characteristics of pregnancy-associated CVST (5–7), evidence specifically focused on early pregnancy CVST—particularly whether women can safely continue their pregnancies—is scarce. A small case series by Wang et al. reported outcomes in 15 patients with first-trimester CVST, but sample sizes were limited and management strategies varied (8).

Given the rarity of pregnancy-associated CVST, single-center retrospective studies remain valuable for generating hypotheses and providing real-world clinical data. Our institution has managed a relatively large cohort of such patients over a 14.5-year period, allowing us to compare early pregnancy CVST with postpartum CVST and to assess long-term outcomes including subsequent pregnancy and recurrence.

To address the above knowledge gaps, this retrospective study aimed to: (1) investigate the risk factors and prognosis of pregnancy-associated CVST by comparing with a control group of pregnant and postpartum women without CVST; (2) describe treatment outcomes, with a particular focus on the prognosis of CVST occurring in early pregnancy; and (3) assess the rates of subsequent pregnancies and CVST recurrence during long-term follow-up (median follow-up duration, 90.6 months; range, 0.2–175.1 months).

## 2 Methods

### 2.1 Study design and participants

We retrospectively reviewed clinical data from 20 consecutive pregnant or postpartum women diagnosed with CVST (CVST group) between January 1, 2010, and June 30, 2024, at Changhai Hospital, a tertiary referral center in Shanghai, China. The diagnosis of CVST was confirmed by at least two experienced specialists—a senior neuroradiologist and a stroke neurologist—based on characteristic findings from neuroimaging. Diagnostic workup followed a standardized institutional protocol and included computed tomography (CT) with CT venography, and/or magnetic resonance imaging (MRI) with magnetic resonance venography (MRV). In diagnostically uncertain cases, digital subtraction angiography (DSA) was performed as the reference standard. No specific exclusion criteria were applied for the CVST group to maximize case capture given the rarity of the condition.

For the control group, 198 pregnant or postpartum women without CVST (non-CVST group) admitted to the Department of Obstetrics and Gynecology at the same hospital during the identical study period (January 1, 2010, to June 30, 2024) were selected using a stratified systematic sampling approach. Specifically, for each calendar year and month within the study period, a list of all non-CVST obstetrical inpatients admitted for delivery or induced abortion was generated from the hospital’s electronic medical record system. From each monthly stratum, eligible controls were uniformly sampled at an approximate ratio of 1:10 relative to the number of CVST cases identified during that corresponding time interval. This stratified sampling strategy was employed to ensure temporal comparability between cases and controls, minimizing potential bias arising from secular trends in clinical practice, diagnostic modalities, or population characteristics over the 14.5-year study period. Controls were not individually matched for demographic or clinical variables but were drawn from the identical source population to preserve baseline comparability regarding healthcare access and institutional referral patterns. Women with a history of CVST or other cerebral venous disorders were excluded from the control group.

### 2.2 Data collection

Comprehensive information was extracted from the electronic medical records, including: (1) patient demographics (age, parity, body mass index [BMI]); (2) pregnancy and obstetric history (gravidity, parity, assisted reproductive technology use, delivery mode); (3) pregnancy-related complications and comorbidities (preeclampsia, gestational diabetes mellitus, hyperemesis gravidarum, postpartum hemorrhage, prior thrombotic events); (4) laboratory parameters {complete blood count, coagulation profile including prothrombin time [PT], activated partial thromboplastin time [APTT], D-dimer, and fibrinogen; serum lipid profile including total cholesterol [TC], triglycerides [TG], high-density lipoprotein cholesterol [HDL-C], low-density lipoprotein cholesterol [LDL-C], apolipoprotein A1 [APO-A1], apolipoprotein B [APO-B], and lipoprotein(a) [Lp(a)]}; (5) neuroimaging findings; and (6) treatment modalities and clinical outcomes. Infections defined as clinical symptoms plus laboratory or imaging evidence, e.g., fever, elevated white blood cell count, or positive imaging findings such as mastoiditis. Good prognosis was defined as modified Rankin Scale (mRS) score ≤ 2 at last follow-up. For patients who could not be retrospectively assessed with mRS, favorable functional outcome was defined as independent daily living without major neurological deficits.

### 2.3 Follow-up

All surviving patients were followed after the index CVST event to ascertain subsequent pregnancy outcomes and any recurrence of CVST. Follow-up was conducted through a combination of reviewing subsequent outpatient clinic records within the hospital’s electronic medical system and structured telephone interviews with patients or their immediate family members. Follow-up was complete for all 16 surviving patients, with the date of last follow-up set at January 1, 2025. Using the reverse Kaplan–Meier method (with death treated as a censoring event), the median follow-up duration for the entire cohort of 20 patients was 90.6 months (interquartile range, 16.7–113.3 months; full range, 0.2–175.1 months). For the 16 surviving patients, the median follow-up duration was also 90.6 months (range, 6.6–175.1 months).

### 2.4 Statistical analysis

Normally distributed continuous variables are presented as mean ± standard deviation (SD), and non-normally distributed variables as median with interquartile range (IQR, 25th–75th percentiles). Between-group comparisons were performed using the independent samples *t*-test or the Mann–Whitney U test, as appropriate. Categorical variables are expressed as frequencies and percentages, with comparisons conducted using the chi-square test or Fisher’s exact test where expected cell counts were < 5.

To identify factors associated with CVST occurrence, variables with a *p* < 0.1 in univariate analysis were entered into a multivariate binary logistic regression model. A forward conditional method was used to build the multivariate model (entry criterion: *p* ≤ 0.05; removal criterion: *p* ≥ 0.10). Due to high collinearity among lipid-related parameters (TC, TG, HDL-C, APO-A1, and APO-B), only APO-A1 was entered into the multivariate model to avoid multicollinearity. Prognostic factors for CVST outcomes were similarly explored using univariate logistic regression. A two-tailed *p* < 0.05 was considered statistically significant. All statistical analyses were performed using IBM SPSS Statistics for Windows, version 24.0 (IBM Corp., Armonk, NY, United States).

### 2.5 Ethical considerations

The study was approved by the Ethics Committee of Naval Medical University and conducted in accordance with the Declaration of Helsinki. Given the retrospective nature of the study, the requirement for written informed consent for data collection was waived by the Ethics Committee. For the telephone follow-up component, verbal informed consent was obtained and documented prior to each interview. For deceased patients, informed consent was waived by the ethics committee. All methods were performed in compliance with relevant guidelines and regulations.

## 3 Results

### 3.1 Basic information of CVST pregnant women

The collected data of pregnant women with CVST are presented in Table 1. Among the cases, 65% (13/20) were primiparas, with 35% (7/20) occurring during pregnancy, all in the early pregnancy stage, while the remaining 65% occurred in the postpartum period. The initial symptoms in early pregnancy were headache, nausea, and vomiting, while the initial symptom in the postpartum period was headache. The shortest time from symptom onset to medical consultation was within half a day (3 h), and the longest was 22 days. At the first consultation, 20% (4/20) of patients have an unclear diagnosis of CVST. Imaging indicated that CVST primarily affected the superior sagittal, transverse, and sigmoid sinus. A 35% (7/20) involved one venous sinus, 20% (4/20) involved two venous sinuses, and 45% (9/20) involved three venous sinuses. Besides, 35% of patients with CVST had concomitant intracerebral hemorrhage, while 45% had cerebral infarction. The incidence of hemorrhage, infarction, and cerebral herniation during pregnancy was significantly higher than in the postpartum period (85.71% versus 7.69%, 57.14% versus 30.77%, and 28.57% versus 7.69%). Among patients with CVST, 45.0% had clinical or imaging evidence of infection, including pneumonia, urinary tract infection, upper respiratory tract infection, mastitis or mastoiditis, and sinusitis). In most cases, 80% were isolated cases of CVST (16/20), while 20% had thrombosis in other locations. Additionally, 15% (3/20) had a prior history of thrombosis. All cases underwent immunological testing with non-significant abnormalities; two cases had abnormal genetic testing results, one indicating autosomal dominant protein S deficiency thrombosis (OMIM: 612336) and autosomal recessive protein S deficiency thrombosis (OMIM: 614514, Diamond-Blackfan anemia type 7 (OMIM: 12562), while the other indicated Hermansky–Pudlak syndrome type 3 (OMIM: 614072). (A summary of key characteristics is presented in Supplementary Table S1).

**Table 1:** Basic information of pregnant and postpartum women with CVST characteristics.

Characteristics	CVST group		
	Pregnancy period (*n* = 7)	Postpartum period (*n* = 13)	Total (*n* = 20)
Parity			
Primipara	5 (71.43%)	8 (61.54%)	13 (65.00%)
Multipara	2 (28.57%)	5 (38.46%)	7 (35.00%)
Initial symptoms			
Headache	3 (42.86%)	9 (69.23%)	12 (60.00%)
Epileptic seizures	0	2 (15.38%)	2 (10.00%)
Limb weakness	1 (14.29%)	0	1 (5.00%)
Nausea/vomiting	3 (42.86%)	1 (7.69%)	4 (20.00%)
Disturbed consciousness	0	1 (7.69%)	1 (5.00%)
Time from symptom initiation to hospital (days)	2–22	0.5–19	/
First consultation confirming CVST	6 (85.71%)	10 (76.92%)	16 (80.00%)
Coexisting thromboses			
CVST	6 (85.71%)	10 (76.92%)	16 (80.00%)
CVST+VTE	1 (14.29%)	2 (15.38%)	3 (15.00%)
CVST+VTE + PE	0	1 (7.69%)	1 (5.00%)
Neuroimaging abnormalities			
Superior sagittal sinus	6 (85.71%)	6 (46.15%)	12 (60.00%)
Sigmoid sinus	4 (57.14%)	7 (53.85%)	11 (55.00%)
Transverse sinus	4 (57.14%)	9 (69.23%)	13 (65.00%)
Straight sinus	1 (14.29%)	1 (7.69%)	2 (10.00%)
Confluence of sinuses	1 (14.29%)	1 (7.69%)	2 (10.00%)
Coexisting intracerebral hemorrhage	6 (85.71%)	1 (7.69%)	7 (35.00%)
Coexisting cerebral infarction	4 (57.14%)	4 (30.77%)	8 (40.00%)
Coexisting brain herniation	2 (28.57%)	1 (7.69%)	3 (15.00%)
Comorbidities			
Previous thrombotic history	1 (14.29%)	2 (15.38%)	3 (15.00%)
Thrombophilia	1 (14.29%)	1 (7.69%)	2 (10.00%)
Infection	3 (42.86%)	6 (46.15%)	9 (45.00%)

PE, Pulmonary embolism; DVT, Deep venous thrombosis.

### 3.2 Incidence and risk factors

We compared the general conditions, comorbidities, complications, and laboratory tests of pregnant and postpartum women with CVST to those without CVST. The results (Table 2) indicated statistically significant differences between the two groups regarding parity, previous thrombotic history, preeclampsia, gestational diabetes, and hyperemesis gravidarum (*p* < 0.05). Among the laboratory indicators, hemoglobin (Hb) and prothrombin time (PT) were significantly higher in the CVST group than in the non-CVST group, with statistically significant differences (*p* < 0.05). However, lipid-related indicators—including total cholesterol (TC), triglycerides (TG), high-density lipoprotein cholesterol (HDL-C), apolipoprotein (APO)-A1, and APO-B—were significantly lower in the CVST group than in the non-CVST group, with statistically significant differences (*p* < 0.05).

**Table 2:** Comparison between pregnant and postpartum women with CVST and normal pregnant and postpartum women.

Clinical characteristics	CVST group (*n* = 20)	Non-CVST group (*n* = 198)	*p*-values
Age (years)	29.44 ± 5.26	30.93 ± 4.99	0.110
Pregnancy history			
Pregnancies	1.00 (1.00, 2.00)	2.00 (1.00, 3.00)	0.305
Deliveries	1.00 (0.00, 1.75)	1.00 (1.00, 1.00)	0.017
History of thrombosis n (%)	3	1	<0.001
BMI (kg·m^2^)	23.71 (21.90, 25.62)	24.46 (23.34, 25.72)	0.559
Pregnancy conditions n (%)			
ART	2	14	0.632
Multiple pregnancies	0	2	0.652
Surgical procedures during pregnancy	0	10	0.304
Pregnancy outcomes n (%)			
Cesarean section	10	84	0.514
preterm birth	2	15	0.700
Stillbirth	1	10	0.992
Pregnancy complications n (%)			
Preeclampsia	2	2	0.004
Gestational diabetes	0	34	0.044
Hyperemesis gravidarum	1	0	0.002
Postpartum hemorrhage	0	0	/
Medical comorbidities	2	5	0.519
Laboratory indicators			
WBC/(×10^9^·L^−1^)	8.96 (5.51, 10.81)	11.01 (8.77, 13.82)	0.041
Neutrophils (%)	72.16 ± 8.86	73.50 ± 8.99	0.920
HB/(g·L^−1^),	120.00 (113.00, 124.75)	108.00 (99.00, 117.00)	0.002
PLT/(×10^9^·L^−1^),	200.50 (164.75, 262.50)	196.00 (155.00, 241.00)	0.747
D-D/(μg·mL^−1^),	3.40 (1.78, 7.10)	1.78 (1.29, 2.42)	0.003
PT/s	13.85 (12.70, 14.90)	12.40 (12.00, 13.10)	<0.001
FIB/(mg·L^−1^),	3.95 (2.53, 19.72)	4.39 (3.86, 4.86)	0.780
APTT/s	33.15 (11.47, 39.95)	33.10 (31.7, 34.50)	0.893
Sodium (mmol/L)	139.50 (134.75, 143.00)	139.00 (137.00, 140.00)	0.768
TC (mmol/L)	4.95 ± 0.89	6.58 ± 1.55	0.002
TG (mmol/L)	1.66 (0.90, 2.49)	2.12 (1.72, 3.35)	0.019
HDL-C (mmol/L)	1.27 (0.94, 1.65)	1.97 (1.61, 2.35)	<0.001
LDL-C (mmol/L)	3.14 (2.63, 3.40)	3.41 (2.76, 4.31)	0.065
APO-A1 (mmol/L)	1.24 ± 0.38	2.16 ± 0.43	<0.001
APO-B (mmol/L)	0.97 ± 0.24	1.33 ± 0.34	0.003
LP a/(mg/dL)	15.00 (6.00, 76.00)	13.00 (9.00, 21.75)	0.568

Gravidity (total number of pregnancies) and parity (number of deliveries) are presented separately to reflect distinct clinical information. BMI, Body Mass Index; ART, Assisted Reproductive Technology; WBC, White Blood Cell Count; HB, Hemoglobin; PLT, Platelet Count; D-D, D-Dimer; PT, Prothrombin Time; FIB, Fibrinogen; APTT, Activated Partial Thromboplastin Time; TC, Total Cholesterol; TG, Triglycerides; HDL-C, High-Density Lipoprotein Cholesterol; LDL-C, Low-Density Lipoprotein Cholesterol; APO-A1, Apolipoprotein A1; APO-B, Apolipoprotein B; LP a, Lipoprotein a.

We conducted a regression analysis on the related risk factors for CVST in pregnant women (Table 3). Univariate analysis indicated that several deliveries, a history of thrombosis, preeclampsia, and differences in Hb, PT, and lipid indicators were associated with CVST in pregnant women (*p* < 0.05). Multivariate analysis indicated that APO-A1 was negatively correlated with the occurrence of CVST in pregnant women and was an independent influencing factor [odds ratio (OR) = 0.005, *p* < 0.05].

**Table 3:** Logistic regression analysis of risk factors associated with CVST in pregnant and postpartum women.

Variable	Univariate analysis		Multivariate analysis	
	OR (95% CI)	*P-*values	OR (95% CI)	*P-*values
Parity	0.320 (0.139 ~ 0.738)	0.008		
History of thrombosis	34.765 (3.428 ~ 352.613)	0.003		
Preeclampsia	10.889 (1.447 ~ 81.964)	0.02		
HB	1.064 (1.018 ~ 1.112)	0.006		
D-D	1.331 (1.113 ~ 1.593)	0.002		
PT	4.015 (2.189 ~ 7.364)	<0.001		
TC	0.301 (0.125 ~ 0.723)	0.007		
TG	0.330 (0.119 ~ 0.916)	0.033		
HDL-C	0.035 (0.004 ~ 0.316)	0.003		
APO-A1	0.004 (0.000 ~ 0.138)	0.002	0.005 (0.000, 0.158)	0.003
APO-B	0.011 (0.000 ~ 0.317)	0.008		

### 3.3 Treatment and prognosis

The treatment and prognosis of CVST in pregnancy and puerperium are shown in Table 4. All pregnant and postpartum women with CVST received anticoagulant therapy. Moreover, 30% (6/20) of patients underwent thrombectomy, thrombolysis, or a combination of both. Among cases of CVST occurring in the postpartum period, 69.23% (9/13) were after cesarean section, which had a higher incidence than vaginal delivery and stillbirth induction (69.23% versus 15.38% versus 7.69%). Overall, 80% (16/20) of patients had a good prognosis after rehabilitation training. However, 20% (4/20) of cases resulted in death, including three cases in early pregnancy (where the pregnancy status was unresolved at the time of death) and one case in the puerperium period. The mortality rate of CVST during pregnancy was significantly higher than that in the puerperium (42.86% versus 7.69%). During the five-year follow-up period, 15% (3/20) of patients with CVST became pregnant again. None of these patients received prophylactic anticoagulation during pregnancy. Among the three patients with subsequent pregnancies, one underwent elective termination, and the other two delivered at term via vaginal delivery without reported obstetric complications and recurrence of CVST. There was a non-significant difference in the rate of subsequent pregnancies between pregnancy-related CVST and puerperium-related CVST (14.29% versus 15.38%).

**Table 4:** The CVST treatment and prognosis.

Characteristics	CVST group		
	Pregnancy period (*n* = 7)	Puerperium period (*n* = 13)	Total (*n* = 20)
Treatment			
Anticoagulation	4 (57.14%)	10 (76.92%)	14 (70.00%)
Anticoagulation + thrombectomy	1 (14.29%)	2 (15.38%)	3 (15.00%)
Anticoagulation + contact thrombolysis	0	1 (7.69%)	1 (5.00%)
Anticoagulation + Thrombectomy + contact thrombolysis	2 (28.57%)	0	2 (10.00%)
Pregnancy outcomes			
Cesarean section	0	9 (69.23%)	9 (45.00%)
Vaginal delivery	0	2 (15.38%)	2 (10.00%)
Induced labor	4 (57.14%)	1 (7.69%)	5 (25.00%)
Miscarriage	0	0	0
Stillbirth induction	0	1 (7.69%)	1 (5.00%)
Death before pregnancy management	3 (42.86%)	/	3 (15.00%)
Prognosis			
Death	3 (42.86%)	1 (7.69%)	4 (20.00%)
Cured	4 (57.14%)	12 (92.31%)	16 (80.00%)
Subsequent pregnancy	1 (14.29%)	2 (15.38%)	3 (15.00%)

Univariate analysis of prognostic risk factors for CVST in pregnant and postpartum women indicated that *p* < 0.1 may suggest potential risk factors, as depicted in Table 5. Among these, pregnancy outcomes were positively correlated with the prognosis of CVST in pregnant and postpartum women (*p* = 0.039).

**Table 5:** Logistic regression analysis of prognostic risk factors for CVST in pregnant and postpartum women.

Variable	Univariate analysis	
	OR (95% CI)	*P-*values
Occurrence time	0.111 (0.009 ~ 1.395)	0.089
Pregnancy outcome	9.662 (1.125 ~ 83.015)	0.039
BMI	2.176 (0.921 ~ 5.140)	0.076
Presence of cerebral hemorrhage	0.121 (0.010 ~ 1.531)	0.103

## 4 Discussion

In this retrospective study of 20 patients with pregnancy-associated CVST, we found that CVST occurring during early pregnancy was associated with a more severe clinical course and higher mortality (42.86%) compared with postpartum CVST (7.69%). Lower serum apolipoprotein A1 (APO-A1) levels showed an association with CVST occurrence (OR = 0.005, 95% CI: 0.000–0.158, *p* = 0.003). However, the wide confidence interval indicates substantial uncertainty due to the small sample size, and this finding requires validation in larger cohorts. During long-term follow-up (median 90.6 months), 18.75% of surviving patients conceived again, and no CVST recurrence was observed. These findings highlight the poor prognosis of early pregnancy CVST and suggest that a prior history of CVST may not be an absolute contraindication to future pregnancy, though individual risk assessment is essential.

It is important to emphasize that, due to the retrospective nature of this study, the observed differences in lipid and coagulation parameters between the CVST and non-CVST groups should be interpreted as associative rather than causal. Whether low HDL-C/APO-A1 or elevated Hb/PT directly promote CVST development, or whether these changes occur secondary to the thrombotic event or reflect underlying pregnancy-related physiological adaptations, cannot be determined from the current data. Prospective studies with serial biomarker measurements are needed to establish temporality.

Pregnancy induces a physiological state resembling Virchow’s triad, characterized by venous stasis, endothelial injury, and hypercoagulability, which collectively predispose women to thrombotic events. The risk of thrombosis increases approximately 5- to 10-fold during pregnancy and up to 20-fold during the puerperium (9). Consistent with previous reports, our study found a higher incidence of CVST during the postpartum period than during pregnancy (5–7). This heightened postpartum risk may be attributable to several factors, including increased venous return, puerperal infection, dehydration, delivery-related trauma, and intracranial hypotension following epidural or spinal anesthesia (10). In our cohort, clinical or imaging evidence of infection was documented in 46.15% of postpartum CVST cases, with mastoiditis being the most common infectious focus. Notably, the prevalence of infection surpassed that of inherited or acquired thrombophilias in this population.

Headache was the predominant initial symptom of postpartum CVST; however, this symptom may be erroneously attributed to post-dural puncture headache following neuraxial anesthesia during cesarean delivery or dismissed as a benign consequence of traditional postpartum confinement practices. In early pregnancy, the initial presentation of headache, nausea, and vomiting frequently overlaps with symptoms of hyperemesis gravidarum, potentially obscuring the underlying diagnosis and delaying appropriate intervention. The median time from symptom onset to hospital presentation in our series reached 22 days, and the diagnosis of CVST was initially missed or delayed 20% of patients. The differential diagnosis for vomiting, seizures, and altered mental status during pregnancy must include not only hyperemesis gravidarum but also eclampsia and Wernicke’s encephalopathy. It is noteworthy that CVST-associated headache is typically diffuse and may be exacerbated by Valsalva maneuvers or recumbency, whereas thunderclap headache is distinctly uncommon (4, 11). This headache pattern likely reflects stretching of dural nerve fibers, elevated intracranial pressure, and associated cerebral parenchymal injury.

Hyperemesis gravidarum has been recognized by the Royal College of Obstetricians and Gynecologists as a risk factor for venous thromboembolism (12), and previous investigations have corroborated an elevated VTE risk among pregnant women with severe vomiting (13). Our data revealed significant differences between the CVST and non-CVST groups in parity, prior thrombotic history, preeclampsia, gestational diabetes mellitus, and hyperemesis gravidarum. Consequently, hyperemesis gravidarum warrants clinical vigilance, and anticoagulant prophylaxis may be considered in selected cases to mitigate thrombotic risk.

It is essential to recognize that pregnancy-associated CVST is biologically heterogeneous. The pathways leading to thrombosis likely differ across clinical subgroups. For instance, in postpartum cases with mastoiditis or sinusitis (46.15% of postpartum CVST in our series), local infection-induced inflammation and adjacent venous inflammation (septic thrombophlebitis) may predominate. In contrast, in early pregnancy cases, hyperemesis gravidarum-associated dehydration and hemoconcentration may play a greater role. Cases with inherited thrombophilia (e.g., protein S deficiency) represent yet another pathway involving constitutive hypercoagulability. By analyzing these subgroups together, our study may obscure pathway-specific risk factors and prognostic differences. Future studies with larger sample sizes should stratify analyses by etiology (infection-associated, thrombophilia-associated, dehydration-associated, etc.) to better understand the distinct biological mechanisms underlying each subtype.

A notable finding of our study was the significantly lower serum lipid profile—including TC, TG, HDL-C, APO-A1, and APO-B—observed in the CVST group compared with controls, coupled with significantly higher Hb and PT levels. Numerous studies have implicated elevated fibrinogen and reduced HDL-C as prothrombotic factors contributing to atherosclerotic and thrombotic diseases (14, 15). HDL-C plays a pivotal role in reverse cholesterol transport, and low HDL-C levels are associated with endothelial dysfunction and a hypercoagulable state (16, 17). Elevated Hb and prolonged PT further paralleled changes typically observed in a prothrombotic milieu and endothelial activation.

Beyond the classical Virchow’s triad, recent advances have highlighted the role of endothelial dysfunction and inflammation as critical drivers of pregnancy-associated thrombosis. During normal pregnancy, the endothelium undergoes physiological adaptations, including increased von Willebrand factor and reduced thrombomodulin expression, creating a prothrombotic state. In CVST, however, additional inflammatory triggers—such as infection (observed in 45% of our cohort), surgical trauma (69.23% of postpartum CVST followed cesarean section), or tissue injury—can further activate the endothelium. This activation leads to upregulation of adhesion molecules (e.g., ICAM-1, VCAM-1), recruitment of leukocytes, and release of procoagulant microparticles, which collectively shift the balance toward thrombosis. The lower HDL-C levels observed in our CVST group may be relevant here, as HDL-C normally exerts anti-inflammatory and endothelial-protective effects via the SR-BI and ABCA1 pathways; low HDL-C may therefore impair endothelial resilience. However, as noted above, causality cannot be inferred from this retrospective study. Future mechanistic studies examining endothelial activation markers (e.g., soluble thrombomodulin, E-selectin) and inflammatory cytokines in pregnancy-associated CVST are warranted.

It should be noted that the confidence interval for the APO-A1 odds ratio was extremely wide (0.000–0.158), reflecting instability in the estimate likely due to the small number of events. Therefore, this finding should be considered exploratory rather than definitive.

In our study, 69.23% (9/13) of postpartum CVST cases followed cesarean delivery, an incidence substantially exceeding that observed after vaginal delivery or stillbirth induction. However, as shown in Table 2, the difference in cesarean section rates between the CVST and non-CVST groups did not reach statistical significance (*p* = 0.514). Previous studies have suggested that cesarean delivery may be associated with an increased risk of CVST due to delivery-associated tissue trauma and endothelial injury, which could trigger the extrinsic coagulation cascade (5, 18, 19). Therefore, regardless of whether cesarean section itself is an independent risk factor, women undergoing cesarean delivery should receive appropriate risk stratification using obstetric VTE scoring systems, vigilant monitoring of clinical symptoms and laboratory parameters (including PT and HDL-C), and timely thromboprophylaxis when indicated.

A critical clinical question concerns whether patients diagnosed with CVST during early gestation can safely proceed with pregnancy. A previous case series of 15 pregnant patients with CVST (gestational age range, 5–12 weeks) reported three deaths, nine survivors (some with residual neurological deficits), and six women who continued their pregnancies, resulting in three vaginal deliveries and three cesarean sections with healthy neonates (8). In our cohort, all four survivors of early pregnancy CVST elected to undergo induced abortion, and two achieved full neurological recovery following rehabilitation. The three deceased patients in this subgroup had unresolved pregnancy status at the time of death. Our data further demonstrate that CVST occurring during pregnancy is associated with a substantially higher case-fatality rate than postpartum CVST (42.86% vs. 7.69%). Moreover, the incidence of intracerebral hemorrhage, cerebral infarction, and cerebral herniation was markedly elevated in the pregnancy group. All three women who died in early pregnancy had concomitant intracerebral hemorrhage. Previous studies have identified infection, seizures, and cerebral hemorrhage as adverse prognostic indicators in pregnancy-associated CVST (20).

Why might CVST occurring in early pregnancy be more severe than postpartum CVST? Several biological differences between early pregnancy and the puerperium warrant consideration. First, the hormonal environment differs dramatically: early pregnancy is characterized by rapidly rising estrogen and progesterone levels, which are known to increase plasma levels of coagulation factors (VIII, von Willebrand factor, fibrinogen) and decrease natural anticoagulants (protein S). In contrast, the postpartum period involves a gradual return to non-pregnant hormonal baselines. Second, vascular adaptation in early pregnancy includes systemic vasodilation and increased venous capacitance, which may exacerbate venous stasis. Third, the immunological state in early pregnancy involves a complex balance of tolerance (to the semi-allogeneic fetus) and maintained innate immune surveillance; dysregulation of this balance could potentially influence thrombotic risk. Fourth, in our cohort, early pregnancy CVST patients had a significantly higher rate of cerebral hemorrhage (85.71% versus 7.69% in postpartum), which may be related to the more acute onset of thrombosis in early pregnancy, leaving less time for collateral venous channel development. We acknowledge, however, that these explanations remain speculative and that our small sample size (*n* = 7 for early pregnancy CVST) precludes definitive conclusions. Larger prospective studies with serial hormonal and vascular function measurements are needed to test these hypotheses.

Whether the ongoing pregnancy itself—through hormonal, hemodynamic, or immunological mechanisms—is associated with more rapid disease progression and an elevated risk of hemorrhagic transformation remains an unresolved question warranting further investigation.

During the long-term follow-up period (median, 90.6 months), 18.75% (3/16) of surviving patients conceived again. None received antepartum anticoagulant prophylaxis, and no CVST recurrence was documented during pregnancy or the puerperium. Among these three patients, one underwent elective termination of pregnancy, while the other two delivered at term via vaginal delivery without obstetric complications. Details on delivery mode and complications have been added to the Results section (see section 2.3). The rate of subsequent pregnancy did not differ significantly between patients with pregnancy-associated and postpartum CVST [14.29% (1/7) vs. 15.38% (2/13)]. Previous systematic reviews have reported CVST recurrence rates of 0.90–3.60% during pregnancy following a prior CVST episode (21–23). The absence of recurrence in our small cohort may be attributable to limited sample size, although it is noteworthy that all cases occurred in the absence of prophylactic anticoagulation.

Based on our limited data (only 3 patients with subsequent pregnancies and no recurrences), as well as previous studies reporting a low recurrence rate with appropriate prophylaxis, a prior history of CVST may not be an absolute contraindication to pregnancy. However, given the small sample size, this conclusion requires cautious interpretation. The risk of recurrence is likely highly individualized, depending on the underlying etiology (e.g., persistent thrombophilia, autoimmune disease, residual venous sinus obstruction). Therefore, rather than routinely discouraging pregnancy, we recommend that each case undergo a thorough pre-pregnancy evaluation by a multidisciplinary team (including neurology, hematology, and maternal-fetal medicine specialists). For subsequent pregnancies, secondary prevention (e.g., risk-stratified anticoagulation) and close multidisciplinary monitoring are strongly advised.

Our study has several limitations. First, the single-center, retrospective design and the small sample size of CVST cases—an inherent challenge when studying rare diseases—limit the generalizability of our findings and the statistical power of our analyses. Second, due to the limited number of events, the multivariate regression model had restricted capacity to adjust for multiple potential confounders; the identified independent association with APO-A1 should therefore be interpreted with caution and requires validation in larger, multicenter cohorts. Third, the absence of a standardized follow-up imaging protocol precludes assessment of long-term venous recanalization rates. Fourth, the control group was not individually matched for potential confounders such as age or gestational age, which may introduce residual bias. Fifth, data on the exact timing of anticoagulation initiation, specific anticoagulant drugs used, and treatment duration were not consistently available in the medical records over the 14.5-year study period, which limits our ability to assess dose–response relationships or treatment-specific effects. Sixth, due to the small sample size, formal subgroup analysis by etiology (e.g., infection-associated vs. thrombophilia-associated vs. dehydration-associated) was not feasible, which limits our ability to assess pathway-specific risk factors and prognostic differences. Despite these limitations, our data highlight a critical clinical dilemma regarding early pregnancy management. Among our cases, the three deceased patients in the first trimester had unresolved pregnancy status at the time of death, and the four survivors opted for pregnancy termination. The decision to continue pregnancy in this specific clinical scenario remains highly challenging. Further research with larger cohorts is needed to define the optimal management strategy and criteria for safely continuing pregnancy in patients diagnosed with CVST in the first trimester, given the high mortality observed in our small series.

## Data Availability

The original contributions presented in the study are included in the article/Supplementary material, further inquiries can be directed to the corresponding authors.
