# Supplementary material for: The hidden dangers of cerebral venous sinus thrombosis: insights into maternal morbidity and mortality during pregnancy
Source: Front Med (Lausanne). 2026 Jul 9;13:1790485. doi: 10.3389/fmed.2026.1790485 (PMC13391897; doi:10.3389/fmed.2026.1790485)
Supplement: Supplementary file 1 [file Table_1.DOCX]

### **Supplementary Table S1. Summary of key characteristics of 20 patients with pregnancy-associated CVST**

| Characteristic | Value |
| --- | --- |
| ****Demographics**** |  |
| Age (years), median (range) | 29.4 (22–41) |
| Primiparous, n (%) | 13 (65.0%) |
| ****Timing of CVST, n (%)**** |  |
| During pregnancy (all first trimester) | 7 (35.0%) |
| Postpartum | 13 (65.0%) |
| ****Initial symptoms, n (%)**** |  |
| Headache | 12 (60.0%) |
| Nausea/vomiting | 4 (20.0%) |
| Seizures | 2 (10.0%) |
| Others | 2 (10.0%) |
| ****Imaging findings, n (%)**** |  |
| Transverse sinus involvement | 13 (65.0%) |
| Superior sagittal sinus involvement | 12 (60.0%) |
| Sigmoid sinus involvement | 11 (55.0%) |
| Intracerebral hemorrhage | 7 (35.0%) |
| Cerebral infarction | 8 (40.0%) |
| ****Management, n (%)**** |  |
| Overall mortality | 4 (20.0%) |
| — During pregnancy (first trimester) | 3/7 (42.9%) |
| — Postpartum | 1/13 (7.7%) |
| Survived | 16 (80.0%) |
| ****Subsequent pregnancy among survivors**** |  |
| Patients with subsequent pregnancy, n (%) | 3/16 (18.8%) |
| CVST recurrence in subsequent pregnancy | 0 (0.0%) |
